# Cobalt-based co-ordination complex-derived nanostructure for efficient oxygen evolution reaction in acidic and alkaline medium

**DOI:** 10.1016/j.heliyon.2022.e10939

**Published:** 2022-10-05

**Authors:** Naveen Kumar, Aashima Sharma, Kritika Rajput, Ramesh Kataria, S.K. Mehta

**Affiliations:** a Department of Chemistry, Panjab University, Sector-14, Chandigarh, India; b Department of Physics, Panjab University, Sector-14, Chandigarh, India

**Keywords:** Cobalt nanostructures, Dehydroacetic acid, Cobalt oxide, O_2_ evolution, Electrocatalyst

## Abstract

Electrochemical water splitting is one of the most important method for energy conversion and storage. For this, the design and development of a low-cost robust electrocatalyst are highly desirable. In this study, Cobalt-based electrocatalyst for Oxygen Evolution Reaction was synthesized by thermal treatment of Cobalt-dehydroacetic acid (Co-DHA). The as-synthesized Co nanostructures and Co-DHA crystals were characterized with powder X-ray diffraction, X-ray photoelectron spectroscopy thermo-gravimetric analysis, and field emission scanning electron microscopy. The electrochemical O_2_ evolution study shows the overpotential (at 10 mV/cm^−2^) correspond to 294 mV vs reference hydrogen electrode (RHE) for K-300 (Co_3_O_4_@300), whereas K-500 (Co_3_O_4_@500) shows 170 mV vs RHE values in 1 M KOH solution, respectively. Similar trends have been observed for electrochemical O_2_ evolution studies in 0.5 M H_2_SO_4_, where K-300 and K-500 shows the overpotential (at 10mV/cm^−2^) of 234 mV vs RHE, and 199 mV vs RHE, respectively. The outcomes show better catalytic efficiency of K-500 as compared to K-300.

## Highlights

• Simple one pot synthesis for precursor of Co_3_O_4_.
• Enhanced activity for oxygen evolution reaction.
• Efficient performance and stability in both acidic and alkaline medium.

## Graphical abstract

**Figure:**
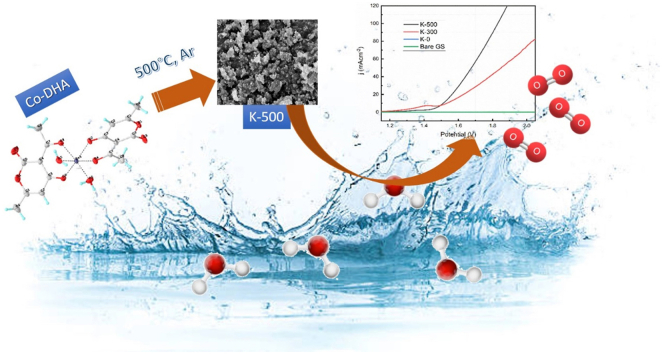

## 1 Introduction

The energy crisis is a broad and complex topic, most of the advanced technologies and innovations are based on carbon-based fossil fuels which are limited in quantity and also cause environmental pollution [1]. To ensure green and sustainable economic growth the exploitation of green and renewable energy sources is the need of hour [2]. For this, the area of gas evolution by the electrochemical water splitting for the use in energy storage, metal-air batteries, fuel cell, etc. has gained much attention [2, 3]. This is because the electrochemical water splitting can produce high-purity Hydrogen (H_2_) which is regarded as a clean fuel with zero carbon emission [4, 5, 6, 7, 8]. The two important reactions involved in the electrochemical water splitting are the Hydrogen evolution reaction (HER) and Oxygen evolution reaction (OER) [9, 10, 11]. However, the kinetics of OER is slightly sluggish and cause hindrance to the water splitting, and limits the efficiency of water splitting [6, 12]. Therefore it becomes highly important to lower the potential barrier of OER and to enhance the efficiency of energy conversion [13, 14]. Precious metal RuO_2,_ IrO_2,_ and Pt-based catalysts are considered efficient electrocatalysts but due to their scarcity and high cost thus the practical use of these catalysts is limited [15, 16].

Therefore, it is important to develop catalysts that eliminate the use of precious metals and increase the efficiency of energy conversion. In the past decade, catalysts developed from transition metal and their derivate which are cost-effective, earth-abundant, good activity and stability have gained much attention [17, 18, 19]. Particularly, the Cobalt (Co) metal-based catalyst has been in main focus, because of its greater stability, good catalytic activity and high efficiency towards OER [20, 21, 22, 23, 24, 25]. Several methods like calcination/annealing, chemical bath deposition, hydrothermal, electrodeposition have been used for the synthesis of cobalt oxide [26, 27, 28, 29, 30]. But calcination/annealing process among all have key advantages like i) tuning the crystallinity with temperature ii) simple process, iv) comparatively cheap and economic process [26]. In literature, there are many reports in which alkaline electrolyte has been used but there are very few reports in which acidic electrolyte has been used for the OER and not much literature available in which both the acidic and alkaline electrolyte has been used for the OER catalysis [24, 31, 32, 33].

Herein, we have synthesized the nanoporous cobalt oxide catalyst by a simple calcination process under the argon (Ar) atmosphere. In which the precursor Co-DHA was prepared by simple one-pot process and crystal of the precursor were obtained by slow evaporation process [34]. Then these crystals were thermally treated to get the catalyst K-500 (Co_3_O_4_@500). The electrode was prepared by drop-casting the catalyst (dissolved in ethanol) on the graphite sheet. This catalyst (K-500) shows low overpotential (10 mAcm^−2^ current density) of 170 mV and 199 mV for the OER in the alkaline (1 M KOH) and acidic (0.5 M H_2_SO_4_) electrolytes respectively. Moreover, the catalyst also shows the good cyclic stability up to 500 cycles.

## 2 Experimental

### 2.1 Material and method

All the chemicals used in this work were obtained from Sigma Aldrich and Avra chemicals and used without any purification. Solvents used were of analytical grade and obtained from Sigma Aldrich and Fischer Scientific Pvt. Ltd. Graphite sheet (GS) having dimension 105 mm × 10 mm× 1.5 mm thick (HSN 3801) and resistance (8–10 Ω) was obtained from Excel Instruments, Maharashtra (India).

### 2.2 Synthesis of catalyst K-500

**Figure 1:**
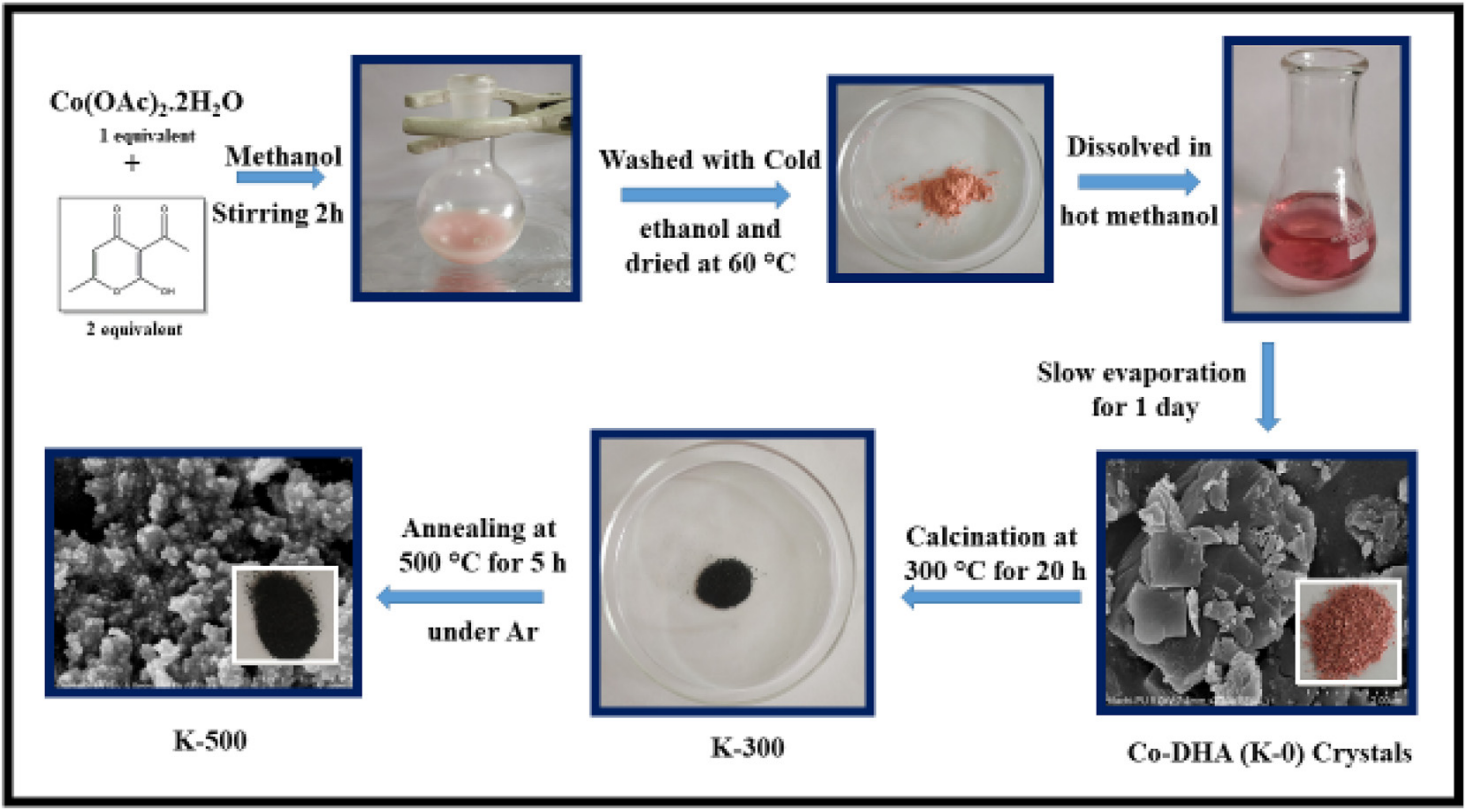
Schematic representation of synthesis of catalyst K-500.

Synthesis of precursor Co-DHA (K-0) was done according to our previously reported method [34] with slight modifications. In a typical synthesis, 2 equivalent DHA was dissolved in 15 mL of methanol (MeOH) in a round bottom flask and stirred with a magnetic stirrer. Then dropwise addition of 1 equivalent Cobalt acetate dissolved in 15 mL methanol was done into the above solution. After stirring for 2 h at 333 K, pink color precipitates were obtained which were filtered, washed with cold ethanol, and dried at room temperature. The solid thus formed was dissolved in hot methanol/ethanol and after one-day crystals of Co-DHA (K-0) were obtained. The as-obtained crystals of K-0 were heated at 573 K and obtained Co nanostructure was denoted as K-300(Co_3_O_4_@300). Further, the K-300 was subjected to heating at 773 K under argon (Ar) atmosphere and obtained Co nanostructure was denoted as K-500. The detailed synthesis procedure of the same has been schematically demonstrated in Figure 1.

### 2.3 Characterization

Powder X-ray diffraction (PXRD) of the prepared samples were carried out in 2θ range of 2°–90° at a scan rate of 7° min^−1^ on a RIGAKU Mini-Flex diffractometer with Cu kα (λ = 0.154 nm) radiation. Surface morphology and elemental composition of the samples have been characterized using a Field Emission Scanning electron microscope (FESEM) equipped with an energy dispersive X-ray analysis (EDX) instrument (Hitachi-SU 8010). The Bruner-Emmett-Teller (BET) surface area was measured by pretreatment of 120 mg of all the samples at 120 °C for 12 h using a BET surface analyzer (BEL Sorp-max) at liquid nitrogen temperature (77 K). The pore size diameter was also obtained with help of the Barret–Joyner–Halender (BJH) method using the adsorption isotherm. The electrochemical measurement was done with the help of three-electrode (Pt as counter, Ag/AgCl (3M KCl) as reference, and Graphite sheet as working electrode) system of Methrohm Multi Autolab (M204).

### 2.4 Preparation of working electrode

The graphite substrate (GS) having dimension 1 cm × 1 cm was washed with isopropyl alcohol, 3 times and dried in the oven at 60 °C. A copper wire was used to make electrical contact with the graphite sheet. A small portion of the GS has been used to drop cast the catalyst and the rest area of the GS was masked using epoxy. The real exposed geometric area of the deposited catalyst over the graphite substrate was 0.5 cm^2^. For the electrochemical measurement, 2.5 mg of each K-0, K-300, and K-500 have been dispersed ultrasonically in the ethanol for 15 min. The as-obtained dispersion was drop cast onto graphite substrate (GS) having geometrical area 0.5 cm^2^ followed by drying at 70 °C for 6 h. The calculated mass loading was 0.62 mg/cm^2^ for each of the catalysts over GS.

## 3 Results and discussion

**Figure 2:**
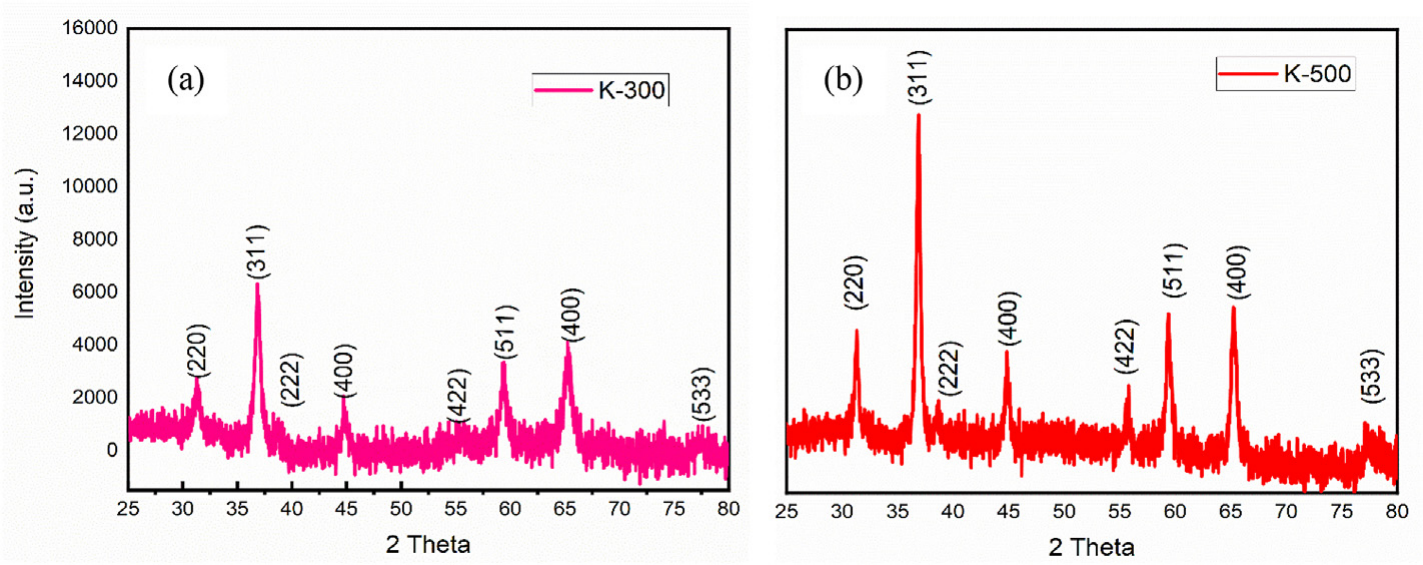
PXRD graphs of (a) K-300 and (b) K-500.

In the present work, we have attempted to synthesize porous cobalt oxide nanostructures from the coordination complex of cobalt (Co) with dehydroacetic acid (DHA) which was prepared by a simple chemical process that was then annealed to get the catalyst K-500 (Figure 2). To know the crystal structure of the prepared porous nanostructures, the Powder X-ray diffraction (P-XRD) patterns of the K-0 (Figure S1), K-500 and K-300 were obtained. Diffraction peaks at 31.37, 36.91, 38.63, 44.81, 55.79, 59.43, 65.30, and 77.23 were observed for K-500 (Figure 2b) and at 31.26, 36.91, 38.73, 44.84, 55.47, 59.43, 65.30 and 77.65 for K-300 (Figure 2a). These peaks were assigned to the (220), (311), (222), (400), (422), (511), (440) and (533) crystal planes respectively. Crystalline nature of all the diffraction peaks for both samples are in good agreement with the JCPDS file of the cubic spinel-type Co_3_O_4_ phase (JCPDS Card No. 74-1656) (Figure 2) [35].

The increased intensity of the P-XRD peaks in K-500 as compared to K-300 shows the increased crystalline nature of Co_3_O_4_.

**Figure 3:**
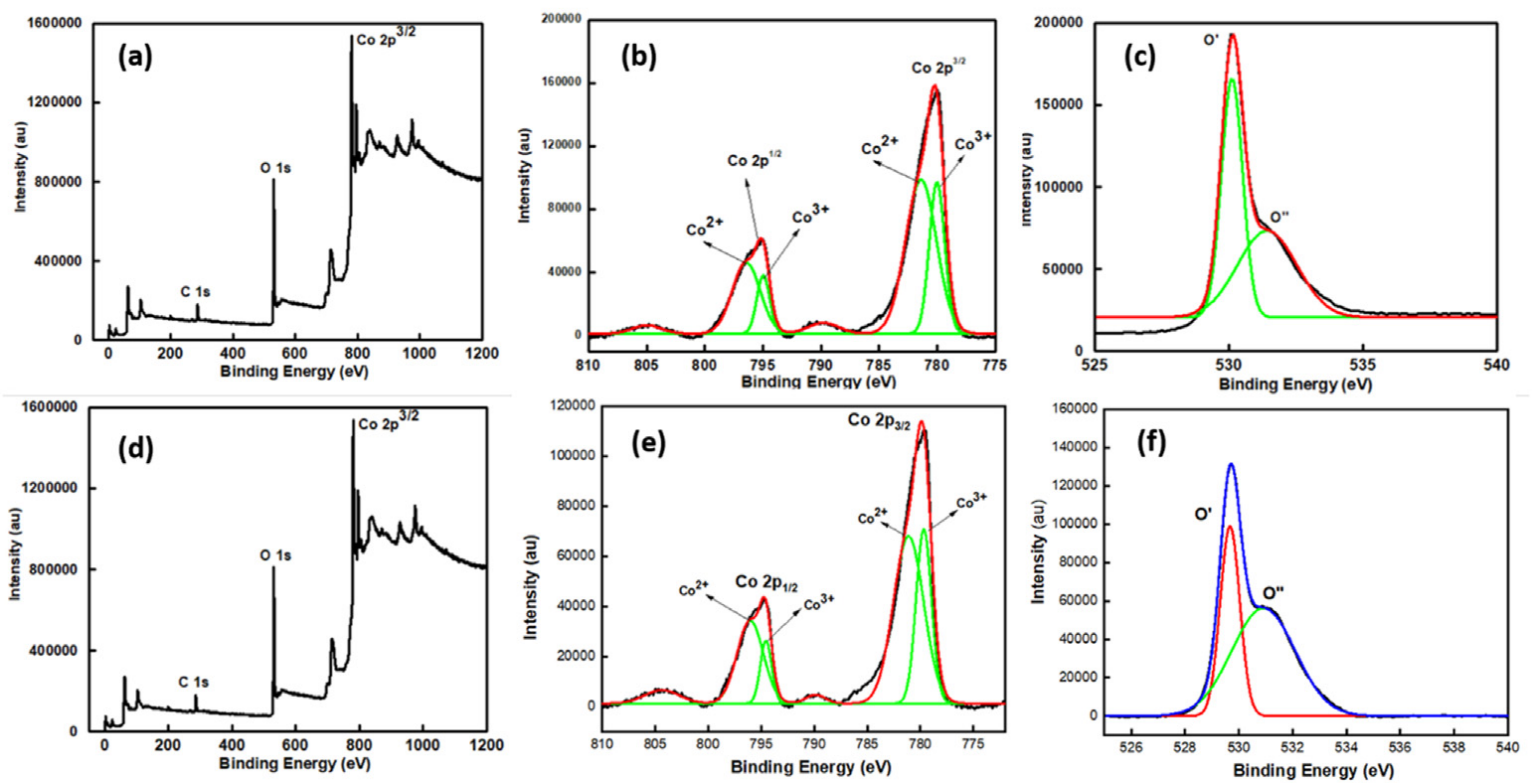
XPS spectra analysis of K-500 (a–c) and K-300 (d–f) with full scan and each metal edge scan.

The XPS spectra depicts the nature of the oxidation state of cobalt in the prepared catalysts K-500 and K-300. Figure 3a represents the XPS survey of the K-500 and Figure 3b signifies the XPS survey of K-300. The peak at 780 eV and a broad satellite peak at around 785.7 eV in the Co 2p_3/2_ region of XPS spectra matches the signals of Co^2+^ species from Cobalt oxide [36]. The deconvoluted spectra of XPS peaks in the Co 2p_3/2_ region shows the evolution of new peaks at 779.9 and 781.34 eV which corresponds to Co^2+^ and Co^3+^ species from Co_3_O_4_ in both K-500 and K-300 (Figure 3 b,e) [37]. The Co^3+^/Co^2+^ ratio found to be 0.72 and 0.88 in case of K-300 and K-500 respectively. The increase in the content of Co^3+^ species suggest the change in the electronic structure which is responsible for enhanced catalytic activity of K-500 [38]. The peak around 530.3 eV is attributed to O^2−^ ion attached to cobalt atoms and peak around 531 eV depicts the chemisorbed O_2_ (Figure 3 c,f) [39]. The peaks represent the nature of the cobalt oxide i.e., mixture of oxidation state is present which is responsible for activity.

**Figure 4:**
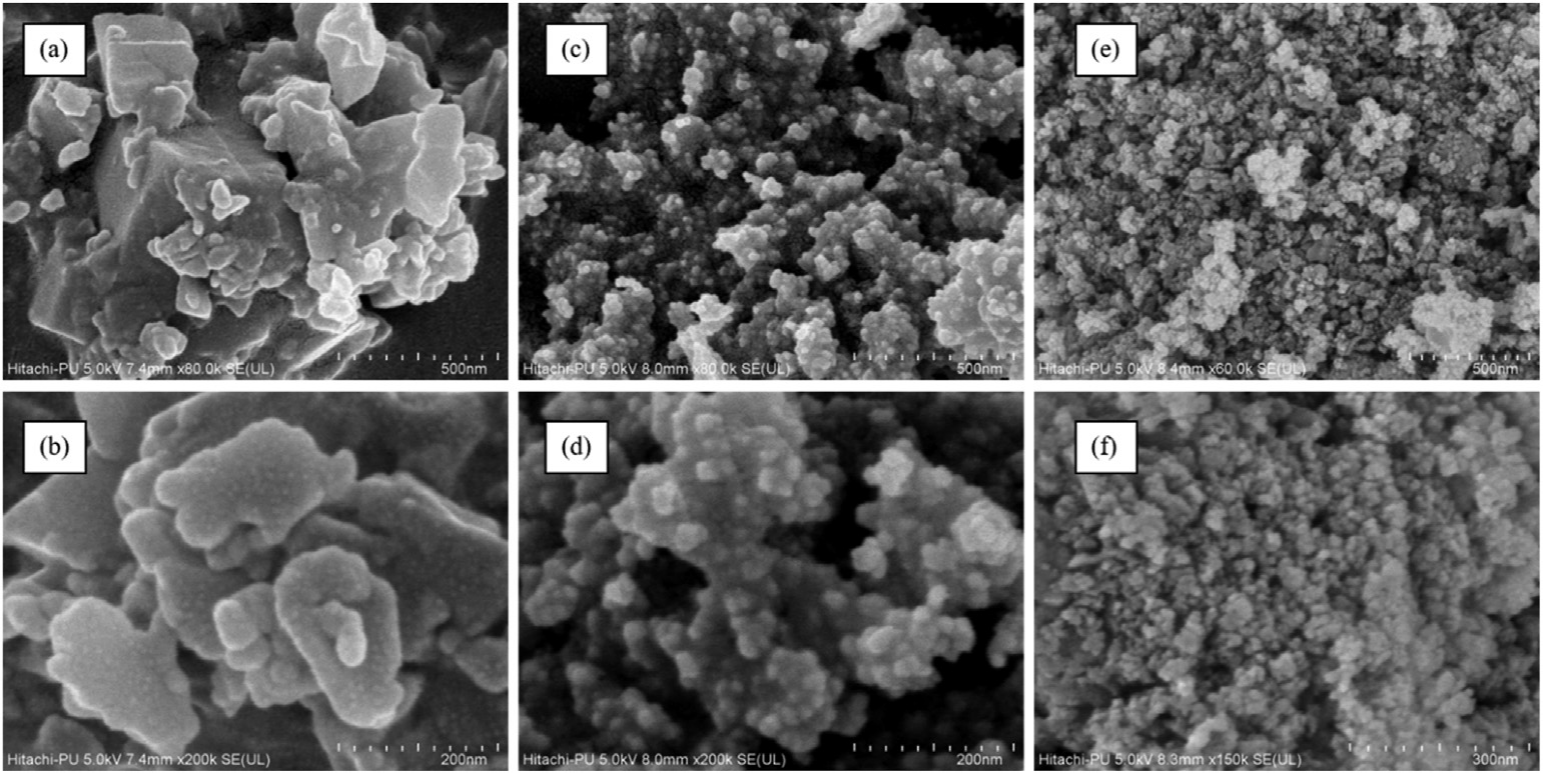
FESEM images (a), (b) of K-0, (c), (d) of K-300, (e) and (f) of K-500 respectively.

**Figure 5:**
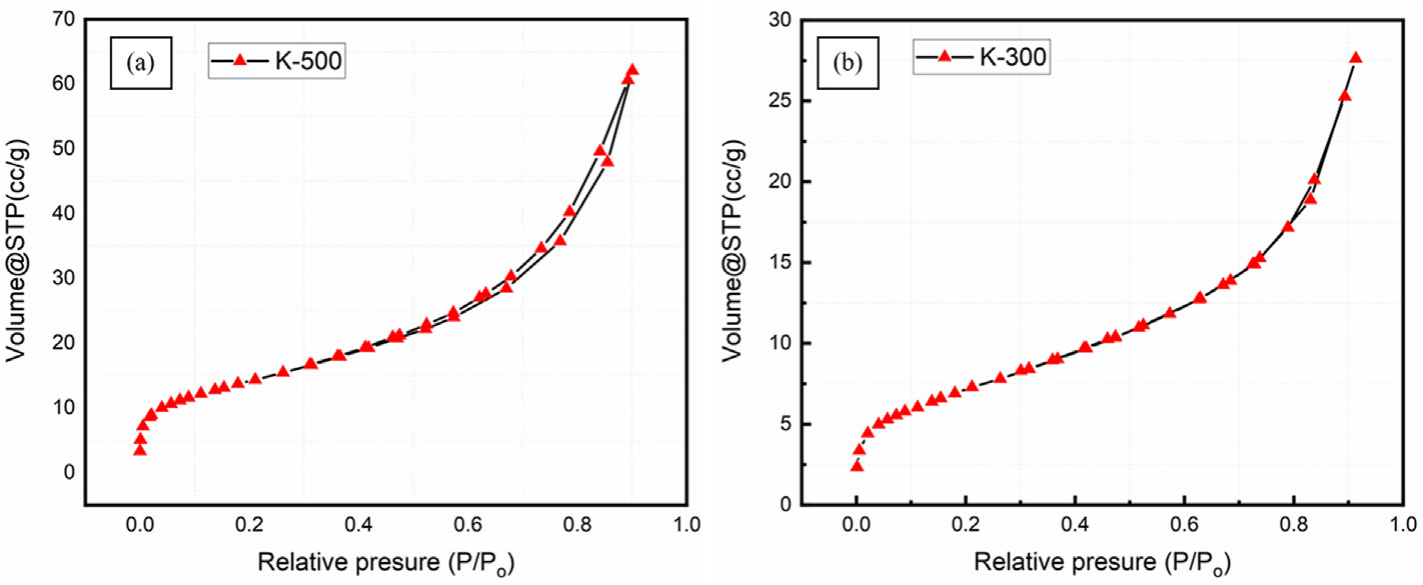
Surface area analysis, Adsorption desorption isotherms of sample (a) K-500 and (b) K-300.

Surface morphology of the Co-DHA, K-300, and K-500 was investigated using Field emission scanning electron (FESEM). Figure 4a and 4b show the FESEM micrographs of the sheets on cubical like K-0 (Co-DHA) complex. Figure 4c and 4d show the FESEM micrographs of K-300, Figure 4e and 4f of K-500 on comparing both the micrographs it is visible that the micrograph of K-500 was annealed at 500 °C under Ar atmosphere have more surface area than K-300 which was annealed at 300 °C. The energy dispersive X-ray spectroscopic analysis (EDS) of K-0, K-300, and K-500 confirms the presence of Co, O, and C (Figure S2a-c). Further, the mapping studies of K-0, K-300, and K-300 shows that all the elements Cobalt, Oxygen, and Carbon are evenly present throughout the sample, Also analyzing the results it is visible that the density of Cobalt metal increased in the order K-0 < K-300 < K-500 (Figure S3a-k). Thermo-gravimetric analysis (TGA) was done to know the temperature stability of the prepared samples K-0, K-300, and K-500. TGA graph of K-0 depicts that the 2 coordinated water molecules were lost at the temperature of 150–200 after which all organic contents were lost and Co_3_O_4_ was the remaining substance in the sample. TGA graphs of K-300 and K-500 shows no significant mass loss which is because Co_3_O_4_ is quite stable up to a wide range of temperature (Figure S4). The Brunauer-Emmett-Teller (BET) characterization was done to evaluate the surface area and pore size distribution (Figure S5) of the samples K-300 and K-500 using nitrogen adsorption-desorption measurements at 77 K, as shown in Figure 5 (a and b) The samples K-500 and K-300 show a specific surface area of 51.55 m^2^/g and 26.81 m^2^/g, respectively.

The corresponding mean pore diameter for the sample K-300 and K-500 was 6.3 A° and 9.6 A°. The isotherm profile confirms the presence of Type IV Langmuir isotherm for both K-300 and K-500. Further, Barrett- Joyner-Halenda (BJH) pore size distribution curve was drawn for each of the samples K-300 and K-500 having pore size varies from 2 to 10 nm. These parameters suggest the mesoporous structure of the samples and this porous structure is beneficial for the electrocatalytic activity. The electrocatalytic activity of the K-0, K-300, and K-500 has been studied in alkaline and acidic electrolyte mediums using a three-electrode system. As earlier discussed in the experimental section, the working electrode has been fabricated by dispersing the catalyst over a graphite sheet. The electrocatalytic activity measurement of K-500 (Co_3_O_4_-500@GS), K-300 (Co_3_O_4_-300@GS) K-0 (Co-DHA@GS), and bare GS electrodes were carried out in anodic polarization potential using electrolyte 1 M KOH and 0.5 M H_2_SO_4_ for O_2_ evolution. The catalytic activity of K-500 and K-300 O_2_ evolution has been attempted.

### 3.1 Oxygen evolution study of the fabricated catalyst

**Figure 6:**
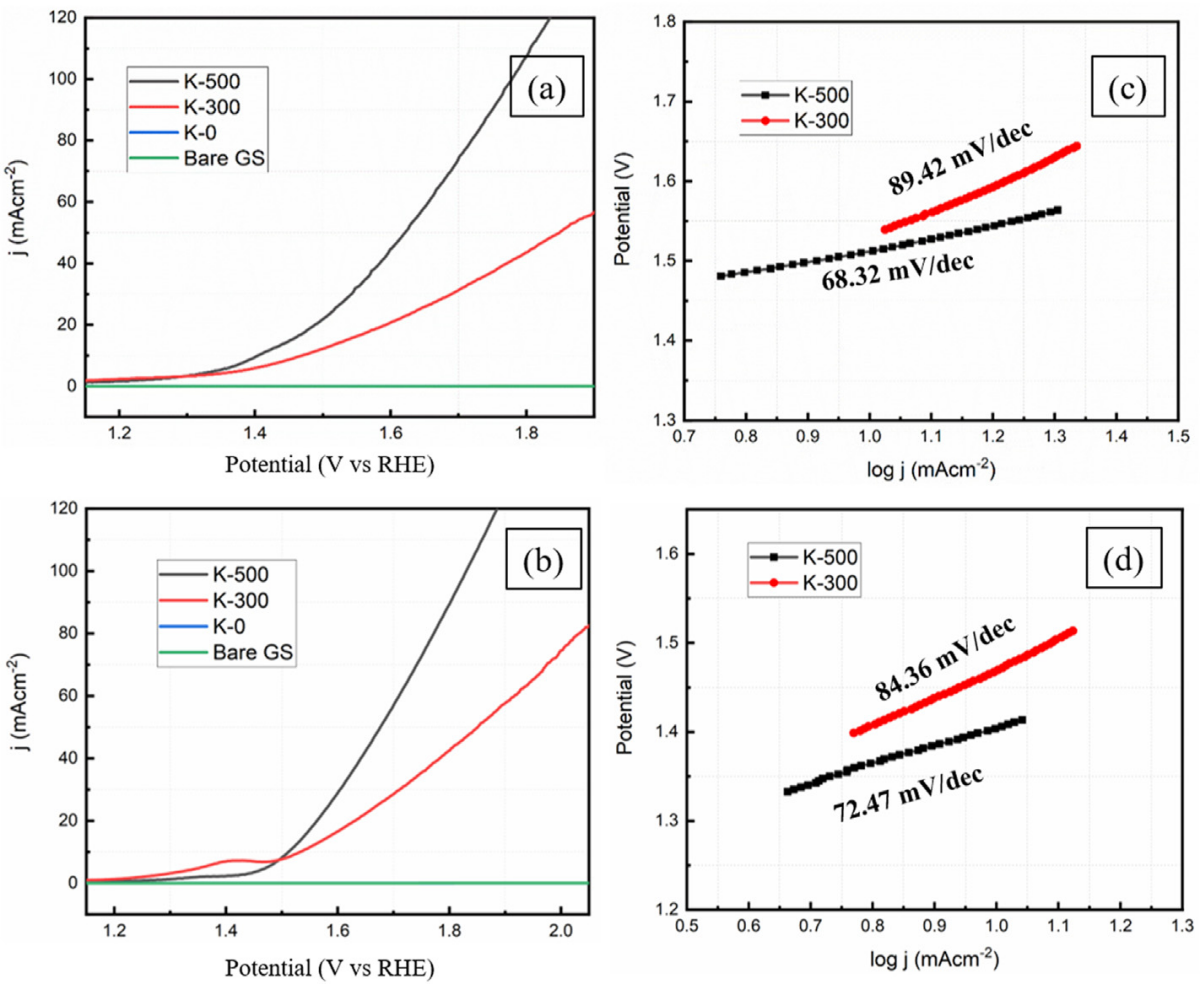
Polarization curves (LSV) of (a) K-500 and K-300 in 1 M KOH, (b) in 0.5 M H_2_SO_4._ Tafel slope of (c) K-500 and K-300 in 1 M KOH, (d) in 0.5 M H_2_SO_4_.

The activity of the catalyst K-500 for oxygen evolution reaction has been done in alkaline (1M KOH) and acidic (0.5 M H_2_SO_4_) electrolyte solution. The linear sweep voltammetry study for K-500 and K-300 was measured at 10 mV/s scan rate (Figure 6a and 6b) the overpotential (ɳ_10_) to reach 10 mA/cm^2^ in 0.5 M in H_2_SO_4_ of the catalyst K-300 and K-500 were 234 mV and 199 mV respectively and in 1M KOH the overpotential of the samples K-300 and K-500 were 294 mV and 170 mV. The observed low overpotential for K-500 confirms the superior catalytic activity than other reported cobalt metal-based catalysts [40, 41, 42, 43, 44]. Shi et. al. has also reported the synthesis of N-doped graphene wrapped pure hexagonal cobalt nanosheets as an electrocatalyst towards oxygen evolution reaction which shows overpotential corresponds to 340 mV [45]. The results confirm the better catalytic efficiency of K-500 than variously reported catalysts **(**Table 1**)**. The Tafel slope measured in 1 M KOH were 68.32 mv/dec, 89.42 mv/dec and in 0.5 M H_2_SO_4_ for K-500, K-300 were 72.47 mv/dec, 84.36 mv/dec, respectively (Figure 6c and 6d). The lower Tafel slope value of the sample K-500 indicates that catalysis proceeded with a much faster kinetic rate compared to sample K-300 O_2_ evolution. The electrochemical impedance spectroscopy (EIS) for K-500, K-300, K-0 and GS shows the linear line nature with R_ct_ values of 27.49 Ω, 34.45 Ω, 63.76 Ω, >5000 Ω respectively (Figure S7).

**Table 1:** Comparison of OER activity and operation condition of synthesized electrocatalyst with those of previously reported catalysts.

S. No	Electrocatalysts	Onset Potential or Overpotential (η_10_) for OER	OER condition	Refs.
1	Pt/LiCoO_2_	285 mV vs RHE	Alkaline electrolyte	[46]
2	Co_x_@CN supported on Ni foam	260 mV vs RHE	1 M KOH	[27]
3.	GC-CoO	340 mV vs RHE	1 M KOH	[47]
4.	Co_3_O_4_/FTO	570 mV vs RHE	0.5 M H_2_SO_4_	[33]
5.	PNC/Co	370 mV vs RHE	1 M KOH	[31]
6.	Ir_0.06_Co_2.94_O	300 mV vs RHE	0.1 M HClO_4_	[48]
7.	Cobalt hydroxide nanosheet	290 mV vs RHE	1 M KOH	[24]
8.	Co–CuO nanoarray	299 mV at 50 mV/s and 330 mV at 100 mV/s vs. RHE	1 M KOH	[6]
9.	K-500	173 mV vs. RHE, 234 mV vs. RHE	1 M KOH 0.5 M H_2_SO_4_	Our Results

**Figure 7:**
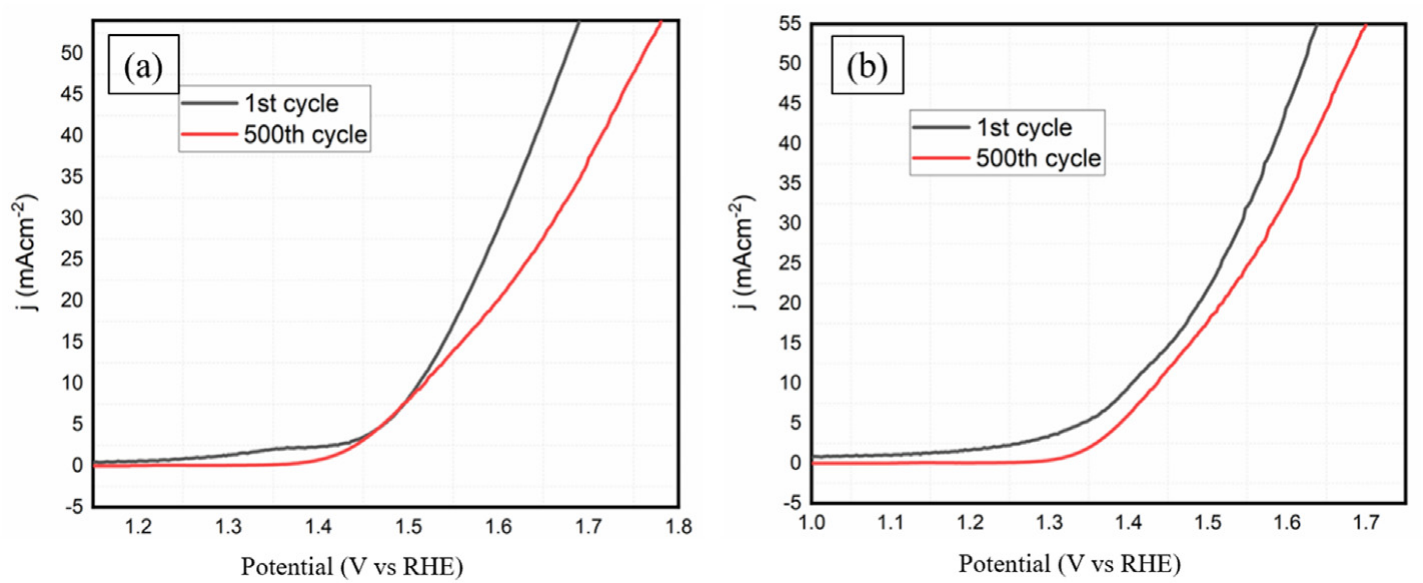
(a) LSV 1st and 500th cycle of K-500 in 0.5 M H_2_SO_4_, (b) in 1 M KOH.

This value indicates the faster and better charge transfer reaction of K-500 as compared to other materials. Further, the stability of the K-500 was measured by performing the LSV for 500 cycles in KOH as well as H_2_SO_4_ electrolyte (Figure 7a, b). And it shows good stability up to 500 cycles with a slight increase in overpotential. As described in the supporting information, the C_dl_ was also calculated for O_2_ evolution in the non-faradic potential region (1.03–1.13 V) (Figure S6). The observed C_dl_ for K-300 and K-500 were 2.32 mF/cm^2^ and 21.07 mF/cm^2^. The evaluation confirms the higher catalytic activity of material K-500 than K-300.

## 4 Conclusion

In the presented work, we have attempted to synthesize Cobalt nanostructure from the coordination complex of cobalt. The cobalt nanostructure was synthesized by thermally treating the Co-DHA complex. Further, the synthesized cobalt nanostructure was drop cost over graphite sheet (GS) used as a working electrode for O_2_ evolution in alkaline as well as acidic medium. In the case of O_2_ evolution, the observed overpotential (at 10mV/cm^−2^) for K-300 and K-500 were 294 mV and 170 mV in alkaline medium, and 199 mV and 234 mV in acidic medium respectively. Further, the catalytic performance for O_2_ evolution confirms the high catalytic activity of K-500 than K-300. The catalytic performance follows a trend as **K-500 > K-300 > K-0 > GS** for O_2_ evolution. Notably, this work presents the synthesis of cobalt nanostructure and the high catalytic performance for O_2_ evolution catalysis as compared to several other reported catalysts (Table 1).

## Declarations

### Author contribution statement

Naveen Kumar: Conceived and designed the experiments; Performed the experiments; Analyzed and interpreted the data; Wrote the paper.

Aashima Sharma: Analyzed and interpreted the data; Wrote the paper.

Kritika Rajput: Performed the experiments.

Ramesh Kataria: Analyzed and interpreted the data.

S.K. Mehta: Analyzed and interpreted the data; Contributed reagents, materials, analysis tools or data.

### Funding statement

This research did not receive any specific grant from funding agencies in the public, commercial, or not-for-profit sectors.

### Data availability statement

Data included in article/supplementary material/referenced in article.

### Declaration of interests statement

The authors declare no conflict of interest.

### Additional information

No additional information is available for this paper.

## Appendix A Supplementary data

Supplementary CoDHA Supplementary CoDHA
