# Supplementary material for: Cobalt-based co-ordination complex-derived nanostructure for efficient oxygen evolution reaction in acidic and alkaline medium
Source: Heliyon. 2022 Oct 5;8(10):e10939. doi: 10.1016/j.heliyon.2022.e10939 (PMC9562245; doi:10.1016/j.heliyon.2022.e10939)
Supplement: Supplementary CoDHA [file mmc1.docx]

Supporting Information

**Cobalt-based co-ordination complex-derived nanostructure for efficient Oxygen Evolution Reaction in Acidic and Alkaline medium**

Naveen Kumar^a^, Aashima Sharma^a^, Kritika Rajput^b^, Ramesh Kataria^a^, S. K. Mehta^a*^.

^a^Department of Chemistry, Panjab University, Sector-14, Chandigarh.

^b^Department of Physics, Panjab University, Sector-14, Chandigarh.

***Corresponding Author**

*Prof. S. K. Mehta*

*Department of Chemistry, Panjab University, Sector-14, Chandigarh*

*Tel: 0172-253 4423*

*Email:* [*skmehta@pu.ac.in*](mailto:skmehta@pu.ac.in)

- 1. **Electrochemical Measurements**

Electrochemical measurement was performed on a three-electrode system at room temperature. In this method, 3M KCl saturated Ag/AgCl was used as a reference and platinum was used as a counter electrode. For O_2_ evolution reaction catalysis, 1 M KOH and 0.5 M H_2_SO_4_ solution has been used as an electrolyte solution. All the potentials were converted to the RHE potential scale by using the Nernst equation as follows.

$E_{RHE}=E_{\frac{Ag}{AgCl}}+0.059\times pH+E_{\frac{Ag}{AgCl}}^{0}$ (1)

Where, $E_{\frac{Ag}{AgCl}}^{0}$ is the standard electrode potential at 25 °C. The overpotential required for the oxygen evolution reaction (OER) is calculated by using the following equation.

$\eta=E_{\frac{Ag}{AgCl}}-1.23 V vs. RHE$ (2)

Here 1.23 V vs RHE is ideal potentials required for the O_2_ evolution. For the electrochemical measurement, Linear Sweep Voltammetry (LSV) was conducted for the samples (K, K-300 and K-500) in the potential range of 1 V-2 V vs RHE for O_2_ evolution measurement in alkaline and acidic media at a 10 mV/s scan rate. Electrochemical impedance spectroscopic (EIS) study of the catalyst was used to determine the ohmic resistance (R_Ω_) in O_2_ catalysis in the frequency range 0.01 Hz -100 kHz at their onset potential with 10 mV amplitude. The FRA software (Nova 1.1) was used for the analysis of the impedance spectrum. The kinetics of catalyst has been evaluated from the Tafel slope using the Tafel equation:

$\eta= a + b log J$ (3)

Where η is overpotential, a is Tafel constant, b is Tafel slope and J is the current density. The C_dl_ was determined from the cyclic voltammetry (CV) scan in the non-faradic potential range at a variable scan rate (10 mV/s, 20 mV/s, 40 mV/s, and 60 mV/s). From the above calculation, current density (mA/cm^2^) at fixed potential vs scan rate (mV/s) was plotted and the value of slope gives the double-layer capacitance (C_dl_).





**Fig. S1.** PXRD graphs of K-0^1^


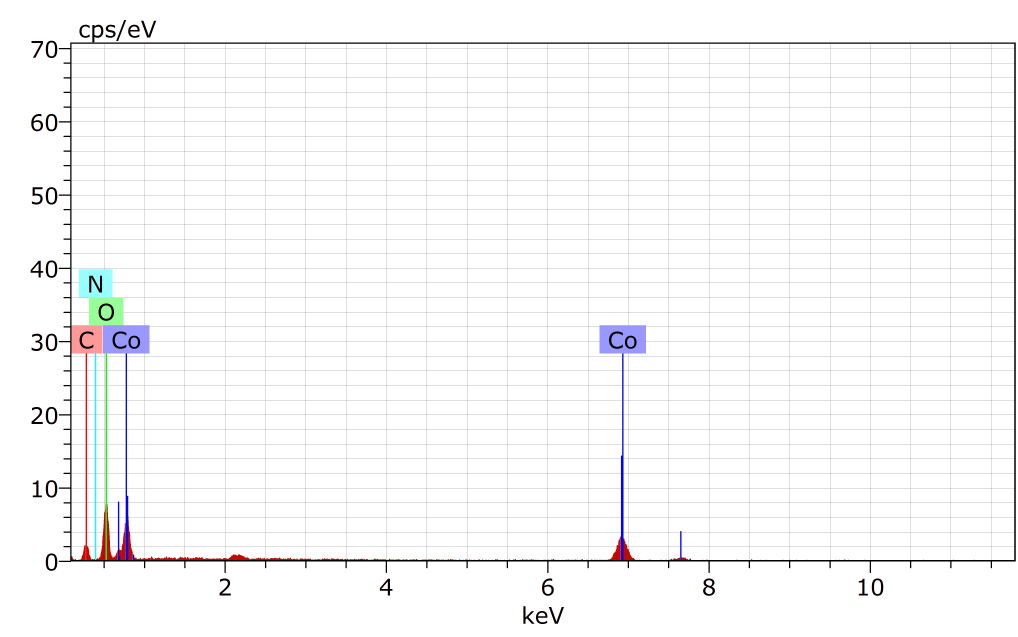


(a)

(b)

**Fig. S2.** EDS spectra shows the percentage of elements Co, C and O present in (a) K-300 and (b) K-500

(a)

(g)

(d)

(c)

(b)

(i)

(f)

(h)

(e)

**Fig. S3.** Mapping images of elements Co, C and O present in (a-c) K-0, (d-f) K-300 and (g-i) K-500 respectively

**Fig. S4.** TGA graph of K-0, K-300 and K-500


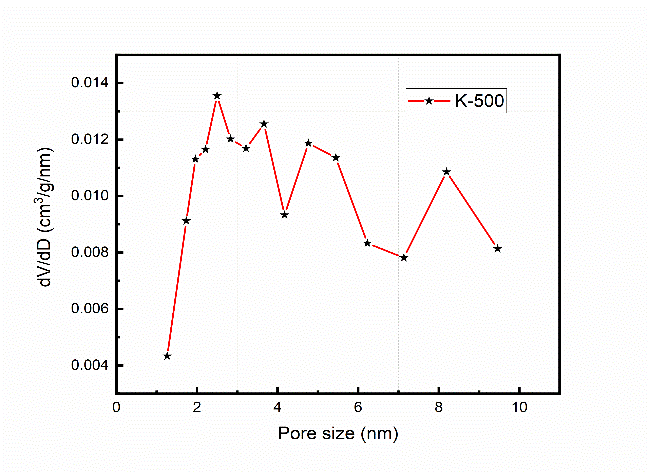

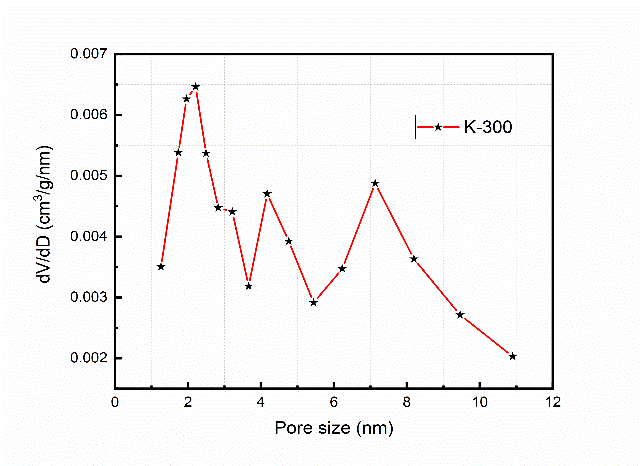


(a)

(b)

**Fig. S5.** Surface area analysis of sample K-300 and K-500 (a, b) corresponding pore size distribution for K-300 and K-500


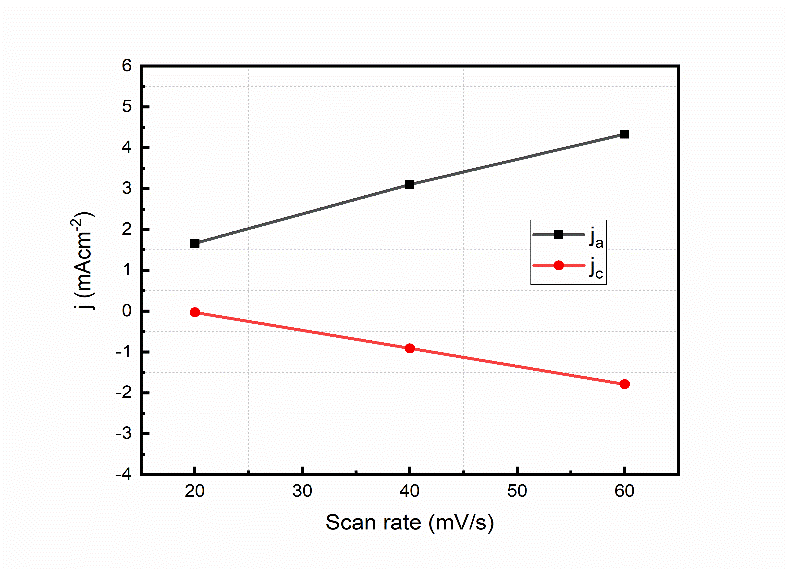

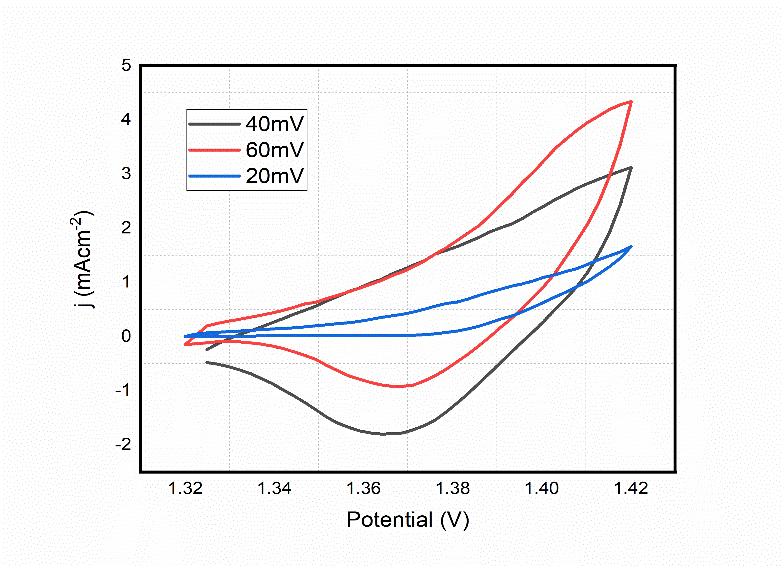


(a)

(b)

**Fig S6**. (a) CV curves measured for C_dl_ calculation and (b) corresponding capacitive current density for Oxygen evolution study of K-500

**

**

**Fig. S7.** (a) Nyquist plot study of different samples

References

1. Wu, H. Bin, Pang, H. & Lou, X. W. Facile synthesis of mesoporous Ni0.3Co2.7O 4 hierarchical structures for high-performance supercapacitors. *Energy Environ. Sci.* **6**, 3619–3626 (2013).
